# Development and validation of an artificial intelligence classifier for histologic grading of superficial non-ampullary duodenal epithelial tumors

**DOI:** 10.1055/a-2868-4714

**Published:** 2026-05-29

**Authors:** Kazuhiro Yamamoto, Atsushi Goto, Shunsuke Ito, Kouichi Hamabe, Shinichi Hashimoto, Jun Nishikawa, Katsuhiko Nakamura, Shu Kiyotoki, Yasuyuki Shirai, Seiji Kaino, Hideo Yanai, Hiroyuki Ogihara, Yoshihiko Hamamoto, Taro Takami

**Affiliations:** 1 Department of Gastroenterology and Hepatology 13150 Yamaguchi UniversityYamaguchiJapan; 2 Department of Laboratory ScienceYamaguchi University Graduate School of MedicineUbeJapan; 3 Department of GastroenterologySaiseikai Shimonoseki General HospitalYanaiJapan; 4 Department of Gastroenterology and Hepatology 310909 Shuto General HospitalYanaiYamaguchi PrefectureJapan; 5 Department of Gastroenterology 13736 Kokura Memorial HospitalKitakyushuJapan; 6 Department of Clinical ResearchNational Hospital Organization Kanmon Medical CenterShimonosekiJapan; 7 Department of Gastroenterology 690960 Hofu Institute of GastroenterologyHofuJapan; 8 National Institute of TechnologyTokuyama CollegeshunanYamaguchiJapan; 9 Sciences and Technology for InnovationYamaguchi University Graduate SchoolUbeJapan

**Keywords:** Endoscopy Small Bowel, Neoplasia, Quality and logistical aspects, Image and data processing, documentatiton, Quality management

## Abstract

**Background and study aims:**

Superficial non-ampullary duodenal epithelial tumors (SNADETs) are being increasingly diagnosed, and accurate endoscopic diagnosis is essential for determining appropriate treatment strategies. However, no standardized diagnostic method currently exists. We developed a deep learning-based artificial intelligence (AI) classifier to distinguish between low-grade and high-grade neoplasia and evaluated its diagnostic performance.

**Patients and methods:**

The training set of 85 SNADET cases (low-grade neoplasia, n = 41; high-grade neoplasia, n = 44; white-light images, n = 317) from Yamaguchi University were used to train the AI classifier. Validation was performed using a test set of 69 lesions (low-grade neoplasia, n = 31; high-grade neoplasia, n =38; images, n = 229) from three institutions. Three expert endoscopists also evaluated the test set using an endoscopic scoring system for comparison.

**Results:**

The AI classifier achieved 78.3% accuracy, 92.1% sensitivity, 61.3% specificity, 74.5% positive predictive value (PPV), 86.4% negative predictive value (NPV), and an F1 measure of 0.824. In contrast, endoscopist diagnosis yielded 58.0% accuracy, 50.0% sensitivity, 67.7% specificity, 65.5% PPV, 52.5% NPV, and an F1 measure of 0.567. Diagnostic accuracy and sensitivity of the AI was significantly higher than that of the endoscopists ( *P* <0.05).

**Conclusions:**

The AI classifier demonstrated higher sensitivity and overall diagnostic performance in comparison to endoscopists in differentiating high-grade neoplasia, suggesting its potential utility in guiding treatment decisions.

## Introduction

Superficial non-ampullary duodenal epithelial tumors (SNADETs) are sporadic neoplasms located in the duodenal mucosa or submucosa and are defined as those not arising from the ampulla of Vater. SNADETs are considered precancerous or early-stage cancerous lesions warranting therapeutic intervention 1 . In Japan, the detection rate for SNADETs has increased in recent years 2 , and with the advancement of endoscopic therapy, endoscopic resection is now recommended as the first-line treatment option 3 .

Accurate endoscopic assessment of histologic grades is essential for informed clinical decision-making. Lesions diagnosed as low-grade neoplasia can be managed with minimally invasive endoscopic procedures such as cold snare polypectomy (CSP) 4 or they may be observed without treatment 5 6 . High-grade malignancies should be resected with electrosurgical techniques, such as endoscopic mucosal resection or endoscopic submucosal dissection 7 . Although multiple endoscopic scoring systems have been proposed to predict histologic grade 8 9 , no standardized diagnostic criteria have been established. Biopsy is less accurate than endoscopy for diagnosing SNADETs and may cause fibrosis, making resection more difficult 9 10 . Therefore, accurate endoscopic diagnosis without a biopsy is preferred for resection of SNADETs 11 12 .

With recent advances in artificial intelligence (AI), its application to diagnosis of gastrointestinal tumors has become feasible. For duodenal lesions, Inoue et al. reported that AI detected SNADETs with 94.7% sensitivity and 87.4% specificity 13 . There is no research describing application of AI in estimation of histological grade from endoscopic images. In this study, we developed a deep learning-based AI classifier to differentiate between low- and high-grade neoplasia in SNADETs and evaluated its diagnostic performance.

## Methods

This study was approved by the Institutional Review Board of Yamaguchi University Hospital (H2022–148), Shuto General Hospital, Kokura Memorial Hospital, National Hospital Organization Kanmon Medical Center and National Institute of Technology, Tokuyama College.

### Subjects

SNADETs that underwent endoscopic resection at Yamaguchi University Hospital between June 2008 and March 2024 were included in the training set. Each lesion was pathologically diagnosed as either low- or high-grade based on the resected specimen following treatment. Low-quality images (e.g., unfocused images or those obscured by blood or mucus) as well as duplicate or similar images were excluded. To prevent overtraining, we limited the number of images to a maximum of six per case. These were captured using one of the following Olympus endoscopes: GIF-H260, GIF-Q260J, GIF-H260Z, GIF-H290, GIF-H290Z, or GIF-EZ1500 (Olympus, Tokyo, Japan). The test set comprised SNADETs resected endoscopically at three participating institutions (Kanmon Medical Center, Kokura Memorial Hospital, and Shuto General Hospital) between September 2009 and February 2023. These were captured using GIF-H260, GIF-Q260J, GIF-H260Z, GIF-H290, GIF-H290Z, or GIF-XZ1200 (Olympus, Tokyo, Japan). Cases associated with familial adenomatous polyposis were excluded from both sets.

All lesions were diagnosed by board-certified gastrointestinal pathologists at each institution. Diagnoses were based on resected specimens obtained via endoscopic treatment and classified according to the Revised Vienna Classification (VCL) for gastrointestinal epithelial neoplasia 14 : category 3, low-grade adenoma; category 4.1, high-grade adenoma; category 4.4, intramucosal carcinoma; and category 5, submucosal invasive carcinoma. Furthermore, it has been reported that neoplasms with gastric characteristics have a higher malignant potential than intestinal-type neoplasms 15 16 . Thus, VCL category 3 intestinal-type lesions were defined as low-grade neoplasia, whereas gastric-type, mixed-type, and VCL category 4/5 lesions were classified as high-grade neoplasia.

### AI classifier development

**Fig. 1:**
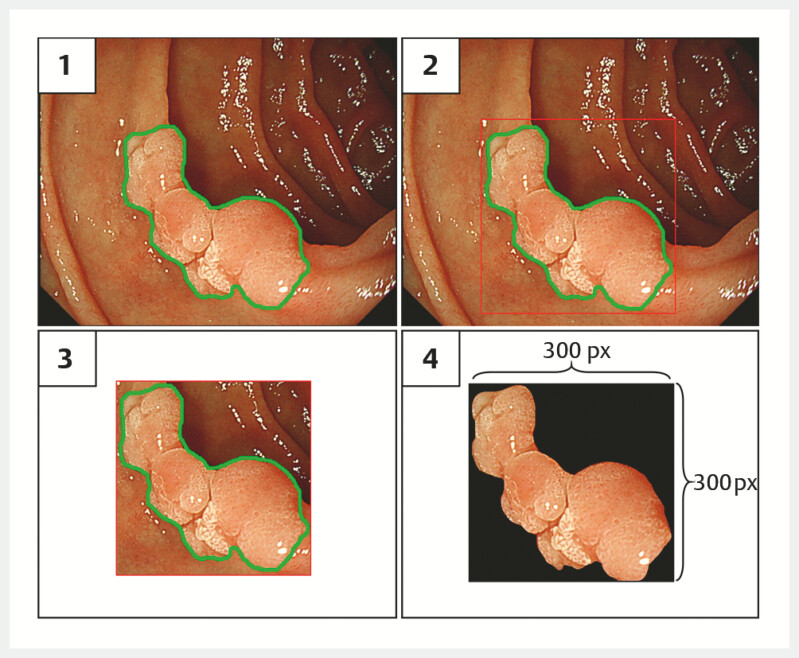
Preprocessing workflow for artificial intelligence (AI) classifier training. Endoscopic images were manually annotated by endoscopists, cropped to isolate the lesion area, masked to exclude background regions, and resized to 300 × 300 pixels. These preprocessed images were then used to train the AI classifier.

The AI classifier was developed in cooperation with National Institute of Technology, Tokuyama College. We used the Efficientnet-B3 model for learning, which was pre-trained on ImageNet, a large dataset of more than 14 million images, to design the AI classifier 17 18 . Two board-certified fellows of the Japan Gastroenterological Endoscopy Society (JGES) annotated tumor regions, which were then enclosed in square bounding boxes, cropped, masked to blacken surrounding areas, and resized to 300 × 300 pixels ( Fig. 1 ). Training was conducted using an NVIDIA GeForce RTX 3070 GPU and an AMD Ryzen Threadripper 3960X CPU.

### Estimation of diagnostic ability using leave-one-out method

Diagnostic ability of the AI classifier to differentiate low- and high-grade neoplasia was estimated by the leave-one-out method 19 with 317 training images. According to this method, the 317 training images were divided into 316 training images and one pseudo-test image. We used the 316 training images to design the AI classifier based on deep learning and diagnosed one pseudo-test image. This step was repeated 317 times such that each of the 317 training images was selected once as a pseudo-test image and diagnostic ability of the AI classifier was evaluated internally.

### AI-based diagnosis: Single- and multi-image analysis evaluations

Although many conventional AI diagnostic systems provide predictions on a per-image basis, we explored a majority vote approach that better aligns with real-world clinical settings. First, prior to the AI analysis, a single “highest-quality image” was designated for each case. This image was chosen in advance by two board-certified fellows of the JGES who performed lesion marking and it was considered to be of the highest quality based on minimal blur, halation, and other artifacts. Next, all images were classified into low- or high-grade tumors using the AI classifier. Subsequently, two distinct analytical methods were established to determine final diagnosis for each case. The first method used AI prediction of the “highest-quality image” as the final diagnosis for the case. We defined this method as “single-image analysis.” The second method determined the final diagnosis based on a majority vote approach, where the most frequent prediction among all images within a case was adopted as the final diagnosis. This method is hereafter referred to as “multi-image analysis.” In situations where the classifications were evenly split, the result from the “highest-quality image” was used instead.

High-grade neoplasia was defined as positive, and accuracy, sensitivity, specificity, positive predictive value (PPV), negative predictive value (NPV), and F1 measure were calculated. The F1 measure is the harmonic mean of sensitivity and PPV, expressed as 2 × sensitivity × PPV/(sensitivity + PPV). We used the F1 measure as a benchmark for making a balanced diagnosis of low- and high-grade neoplasia.

### Comparison with endoscopist diagnosis

Three endoscopists (board-certified fellows of the JGES) evaluated the 69 test lesions using an endoscopic scoring system 8 that assigned 0 to 2 points each for lesion diameter, color, macroscopic type, and nodularity. Lesions scoring ≤2 points were classified as VCL category 3 and those scoring ≥3 as VCL category ≥4. Median score among the three endoscopists was adopted as the final consensus diagnosis of endoscopists. Diagnostic performance for each test case was measured by accuracy, sensitivity, specificity, PPV, NPV, and F1 measure, consistent with the AI-based diagnosis. Interobserver agreement among three endoscopists was evaluated using the Fleiss’ kappa (κ) coefficient.

### Statistical analysis

McNemar’s test was employed to compare diagnostic performance between the different AI models, as well as between AI and endoscopists. Specifically, individual tests were performed to evaluate accuracy, sensitivity, and specificity for each comparison.

## Results

### Clinicopathological characteristics

Table 1 and Table 2 summarize characteristics of training and test lesions. Among training lesions, 85 lesions from 80 patients (41 low-grade and 44 high-grade) were analyzed, with up to six white light images per lesion collected, for a total of 317 images. Among test lesions, 69 lesions from 66 patients (31 low-grade and 38 high-grade) were analyzed, with up to six white light images per lesion collected, for a total of 229 images.

**Table:** **Table 1** Clinicopathological characteristics of training lesions (cases, n = 85; images, n = 317).

	Low-grade neoplasia (41 lesions, 136 images)	High-grade neoplasia (44 lesions, 181 images)
Median age, years (range)	66 (38–84)	69 (37–83)
Male/female, n (%)	30 (73%)/11 (27%)	33 (75%)/11 (25%)
Location of the lesion, n (%)		
Superior part/descending and horizontal part	6 (15%)/35 (85%)	12 (27%)/32 (73%)
Median tumor diameter, mm (range)	5 (3–15)	13 (2–30)
Macroscopic type, n		
0-I/0-IIa/0-IIb/0-IIc	2/27/1/11	7/29/0/8
Histology, n		
VCL category 3/4.1/4.4/5	41/0/0/0	0/11/33/0
Intestinal/mixed/gastric type	41/0/0	38/2/4
Underwent biopsy, n (%)	32 (76%)	36 (82%)
VCL, Revised Vienna Classification		

**Table:** **Table 2** Clinicopathological characteristics of test lesions (cases, n = 69; images, n = 229).

	Low-grade neoplasia (31 lesions, 91 images)	High-grade neoplasia (38 lesions, 138 images)
Median age, years (range)	64 (47–87)	69 (46–92)
Male/female, n (%)	25 (81%)/6 (19%)	28 (74%)/10 (26%)
Location of the lesion, n (%)		
Superior part/descending and horizontal part	5 (16%)/26 (84%)	14 (37%)/24 (63%)
Median tumor diameter, mm (range)	6 (2–20)	11 (4–25)
Macroscopic type, n		
0-I/0-IIa/0-IIb/0-IIc	8/17/1/5	15/20/0/3
Histology, n		
VCL category 3/4.1/4.4/5	31/0/0/0	0/16/18/4
Intestinal/mixed/gastric type	31/0/0	37/0/1
Underwent biopsy, n (%)	24 (77%)	34 (90%)
VCL, Revised Vienna Classification.		

### Diagnostic performance with training set

Table 3 shows AI classifier performance with the training images using the leave-one-out method. Results of single-image analysis with training images were as follows: accuracy, 80.0%; sensitivity, 86.4%; specificity, 73.2%; PPV, 77.6%; NPV, 83.3%; and F1 measure, 0.817. Results of multi-image analysis with training images were as follows: accuracy, 82.4%; sensitivity, 84.1%; specificity, 80.5%; PPV, 82.2%; NPV, 82.5%; and F1 measure, 0.831.

**Table:** **Table 3** Diagnostic performance with the training set (leave-one-out method).

	Accuracy	Sensitivity	Specificity	PPV	NPV	F1 measure
Single image	80.0%	86.4%	73.2%	73.6%	83.3%	0.817
Multi-image	82.4%	84.1%	80.5%	82.2%	82.5%	0.831
NPV, negative predictive value; PPV, positive predictive value.						

### Diagnostic performance with the test set

Table 4 shows AI classifier performance with test images. Single-image analysis results with test images were as follows: accuracy, 75.4%; sensitivity, 86.8%; specificity, 61.3%; PPV, 73.3%; NPV, 79.2%; and F1 measure, 0.795. Results with multi-image analysis of test images were as follows: accuracy, 78.3%; sensitivity, 92.1%; specificity, 61.3%; PPV, 74.5%; NPV, 86.4%; and F1 measure, 0.824.

**Table:** **Table 4** Diagnostic performance with test set.

	Accuracy	Sensitivity	Specificity	PPV	NPV	F1 measure
Single image	75.4%	86.8%	61.3%	73.3%	79.2%	0.795
Multi-image	78.3%	92.1%	61.3%	74.5%	86.4%	0.824
NPV, negative predictive value; PPV, positive predictive value.						

### Endoscopist performance

Table 5 shows endoscopist performance using a scoring system. Results were as follows: accuracy, 58.0%; sensitivity, 50.0%; specificity, 67.7%; PPV, 65.5%; NPV, 52.5%; and F1 measure, 0.567. The Fleiss’ kappa coefficient among the three observers (A, B, and C) was 0.166.

**Table:** **Table 5** Diagnostic performance of endoscopists (scoring system).

	Accuracy	Sensitivity	Specificity	PPV	NPV	F1 measure
Dr. A	59.4%	63.2%	54.8%	63.2%	54.8%	0.632
Dr. B	65.2%	55.3%	77.4%	75.0%	58.5%	0.636
Dr. C	58.0%	47.4%	71.0%	66.7%	52.3%	0.554
Median score	58.0%	50.0%	67.7%	65.5%	52.5%	0.567
NPV, negative predictive value; PPV, positive predictive value.						

### Comparison of diagnostic performance with McNemar’s test

**Fig. 2:**
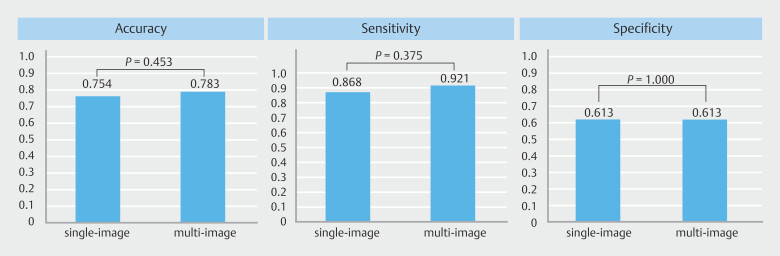
Results of McNemar’s test on diagnostic performance of artificial intelligence between single-image analysis and multi-image analysis

**Fig. 3:**
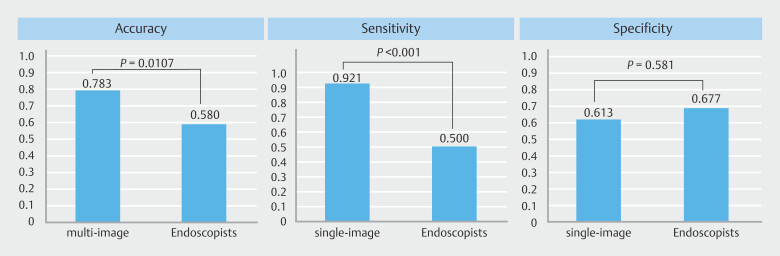
Results of McNemar’s test on the diagnostic performance of artificial intelligence (multi-image analysis) and endoscopists.

Fig. 2 shows results of McNemar’s test comparing diagnostic performance of the AI between single- and multi-image analyses. No significant differences were observed in diagnostic accuracy, sensitivity, or specificity ( *P* >0.05). Fig. 3 presents results of McNemar’s test comparing diagnostic performance of AI (multi-image) and endoscopists. AI accuracy was significantly higher than that of endoscopists (78.3% vs. 58.0%, *P* <0.05). Similarly, a significant difference was observed in sensitivity (92.1% vs. 50.0%, *P* <0.05), whereas no significant difference was found in specificity (61.3% vs. 67.7%, *P* = 0.581).

## Discussion

Our AI classifier demonstrated performance suitable for clinical application with 78.3% accuracy, 92.1% sensitivity, 61.3% specificity, and an F1 measure of 0.824 with test cases collected from three different facilities, which were different from the training cases. To our knowledge, this is the first study to assess AI-based classification of histological grade of SNADETs. These results suggest that model architecture was appropriate and training diversity was sufficient. Although our analysis revealed no statistically significant difference between single- and multi-image approaches, we determined that the multi-image approach based on majority voting is more clinically relevant. This approach mitigates potential for subjective selection bias that occurs when an endoscopist manually selects the “highest-quality image”. By utilizing a comprehensive set of images, we have established a more objective and robust foundation for the AI diagnostic process. Furthermore, we considered that this approach allows for comparisons under clinically representative conditions because endoscopists typically rely on multiple images per case to reach a diagnosis.

In Japan, endoscopic resection is widely recommended for SNADETs 3 , highlighting the critical role of accurate endoscopic diagnosis in clinical decision-making. Duodenal endoscopic procedures are technically demanding and associated with a high risk of adverse events (AEs). Electrocautery-based techniques, including endoscopic submucosal dissection and endoscopic mucosal resection, are particularly prone to complications such as perforation and bleeding 20 . In contrast, CSP is technically simpler and associated with a lower incidence of AEs 20 21 . However, CSP is unable to achieve full submucosal excision, which may limit accuracy of histological evaluation at the resection margins. Consequently, CSP is only considered appropriate for lesions that have been diagnosed as low-grade neoplasms preoperatively, corresponding to VCL category 3 4 . Furthermore, it may be acceptable not to perform resection for low-grade adenomas measuring < 10 mm 5 6 , suggesting that reliable pretreatment grading could help minimize unnecessary interventions. By contrast, lesions categorized as VCL 4 or 5 warrant resection and electrocautery-assisted techniques have been shown to reduce local recurrence rates 7 . Taken together, accurate endoscopic differentiation between low- and high-grade neoplasms is essential for optimizing clinical management, enabling appropriate selection of patients for minimally invasive treatment or surveillance.

The AI classifier outperformed expert endoscopists using the endoscopic scoring system 8 and demonstrated higher sensitivity and consistency. Notably, interobserver agreement among endoscopists was low (κ = 0.166), suggesting that AI can provide more reproducible judgments. Although biopsies also can be used to make a diagnosis, previous studies have reported that biopsies show lower sensitivity (58.0%) in SNADETs relative to endoscopy 22 , and post-biopsy fibrosis can hinder endoscopic resection 10 . Currently, routine biopsy is not recommended when endoscopic treatment is anticipated, highlighting the importance of accurate endoscopic diagnosis. We believe that incorporating our AI classifier into the diagnostic workflow may help prevent overlooking tumors with higher malignant potential.

Although our AI classifier was trained solely on white light imaging, using additional imaging modalities may enhance its diagnostic performance. In current clinical practice, diagnosis of SNADETs commonly involves white light image in combination with indigo carmine staining, narrow-band imaging, and magnified narrow-band imaging observations. For this AI system as well, integrating multiple modalities may further improve diagnostic accuracy. Therefore, additional research is needed to explore multimodal AI-based approaches.

This study has some limitations. The number of training images was small and it came from a single institution. In general, AI models exhibit degradation in performance when evaluated on unknown test data compared with the training phase. However, despite using test cases from three independent institutions that were entirely distinct from the training set, the decrease in performance was negligible. Specifically, F1 measure was 0.831 during training and remained as high as 0.824 during testing in multi-image analysis AI. Future models should be trained on more diverse, multicenter training images to ensure generalizability. In addition, only a few gastric-type lesions were included despite their known aggressiveness and poor prognosis 15 16 . Additional data collection and validation will be essential to improve model generalizability and ensure reliable performance across diverse clinical settings. This study focused specifically on distinguishing low- from high-grade tumors. In addition, our AI was designed to focus on histological grading within tumors rather than initial differentiation between tumor and non-tumor lesions. Although this narrower scope was chosen to address clinical difficulty of grading SNADETs, incorporating non-tumor cases remains a task for future development.

## Conclusions

Our AI classifier achieved favorable diagnostic performance in classifying histological grade of SNADETs and may contribute to optimized treatment selection and clinical decision-making.
